# A Strategy Utilizing Protein–Protein Interaction Hubs for the Treatment of Cancer Diseases

**DOI:** 10.3390/ijms242216098

**Published:** 2023-11-08

**Authors:** Nicolas Carels, Domenico Sgariglia, Marcos Guilherme Vieira Junior, Carlyle Ribeiro Lima, Flávia Raquel Gonçalves Carneiro, Gilberto Ferreira da Silva, Fabricio Alves Barbosa da Silva, Rafaela Scardini, Jack Adam Tuszynski, Cecilia Vianna de Andrade, Ana Carolina Monteiro, Marcel Guimarães Martins, Talita Goulart da Silva, Helen Ferraz, Priscilla Vanessa Finotelli, Tiago Albertini Balbino, José Carlos Pinto

**Affiliations:** 1 Platform of Biological System Modeling, Center of Technological Development in Health (CDTS), Oswaldo Cruz Foundation (FIOCRUZ), Rio de Janeiro 21040-900, RJ, Brazil; carlyle.lima@fiocruz.br (C.R.L.); gilberto.silva@fiocruz.br (G.F.d.S.); 2 Engenharia de Sistemas e Computação, Instituto Alberto Luiz Coimbra de Pós-Graduação e Pesquisa de Engenharia (COPPE), Federal University of Rio de Janeiro (UFRJ), Rio de Janeiro 21941-972, RJ, Brazil; domenico@ufrj.br; 3 Computational Modeling of Biological Systems, Scientific Computing Program (PROCC), Oswaldo Cruz Foundation (FIOCRUZ), Rio de Janeiro 21040-900, RJ, Brazil or marcosvieira.research@gmail.com (M.G.V.J.); fabricio.silva@fiocruz.br (F.A.B.d.S.); 4 Center of Technological Development in Health (CDTS), Oswaldo Cruz Foundation (FIOCRUZ), Rio de Janeiro 21040-900, RJ, Brazil; flavia.carneiro@fiocruz.br (F.R.G.C.); rafaelascardini@edu.unirio.br (R.S.); 5 Laboratório Interdisciplinar de Pesquisas Médicas, Instituto Oswaldo Cruz (IOC), Oswaldo Cruz Foundation (FIOCRUZ), Rio de Janeiro 21040-900, RJ, Brazil; 6 Program of Immunology and Tumor Biology, Brazilian National Cancer Institute (INCA), Rio de Janeiro 20231-050, RJ, Brazil; 7 Centro de Ciências Biológicas e da Saúde (CCBS), Universidade Federal do Estado do Rio de Janeiro (UNIRIO), Rio de Janeiro 22290-255, RJ, Brazil; 8 Dipartimento di Ingegneria Meccanica e Aerospaziale (DIMEAS), Politecnico di Torino, 10129 Turin, Italy; jackt@ualberta.ca; 9 Department of Data Science and Engineering, The Silesian University of Technology, 44-100 Gliwice, Poland; 10 Department of Physics, University of Alberta, Edmonton, AB T6G 2J1, Canada; 11 Department of Pathology, Instituto Fernandes Figueira, Oswaldo Cruz Foundation (FIOCRUZ), Rio de Janeiro 22250-020, RJ, Brazil; cecilia.andrade@fiocruz.br; 12 Laboratory of Osteo and Tumor Immunology, Department of Immunobiology, Fluminense Federal University, Rio de Janeiro 24210-201, RJ, Brazil; anacarolinadossantosmonteiro@id.uff.br; 13 Chemical Engineering Program, Alberto Luiz Coimbra Institute for Graduate Studies and Research in Engineering (COPPE), Federal University of Rio de Janeiro (UFRJ), Rio de Janeiro 21941-594, RJ, Brazil; mmartins@peq.coppe.ufrj.br (M.G.M.); talitasilva@peq.coppe.ufrj.br (T.G.d.S.); helen@peq.coppe.ufrj.br (H.F.); pinto@peq.coppe.ufrj.br (J.C.P.); 14 Laboratório de Nanotecnologia Biofuncional, Departamento de Produtos Naturais e Alimentos, Faculdade de Farmácia, Federal University of Rio de Janeiro (UFRJ), Rio de Janeiro 21941-902, RJ, Brazil; finotelli@pharma.ufrj.br; 15 Nanotechnology Engineering Program, Alberto Luiz Coimbra Institute for Graduate Studies and Research in Engineering (COPPE), Federal University of Rio de Janeiro (UFRJ), Rio de Janeiro 21941-594, RJ, Brazil; tiagoab@pent.coppe.ufrj.br

**Keywords:** tumors, hubs, interactome, RNA-seq, attractors, drug repurposing, RNAi, tumor on a chip, in vivo validation, clinical trial

## Abstract

We describe a strategy for the development of a rational approach of neoplastic disease therapy based on the demonstration that scale-free networks are susceptible to specific attacks directed against its connective hubs. This strategy involves the (i) selection of up-regulated hubs of connectivity in the tumors interactome, (ii) drug repurposing of these hubs, (iii) RNA silencing of non-druggable hubs, (iv) in vitro hub validation, (v) tumor-on-a-chip, (vi) in vivo validation, and (vii) clinical trial. Hubs are protein targets that are assessed as targets for rational therapy of cancer in the context of personalized oncology. We confirmed the existence of a negative correlation between malignant cell aggressivity and the target number needed for specific drugs or RNA interference (RNAi) to maximize the benefit to the patient’s overall survival. Interestingly, we found that some additional proteins not generally targeted by drug treatments might justify the addition of inhibitors designed against them in order to improve therapeutic outcomes. However, many proteins are not druggable, or the available pharmacopeia for these targets is limited, which justifies a therapy based on encapsulated RNAi.

## 1. Introduction

Cancer presents one of the greatest challenges in the medical field globally due to its intricate link to the physiological imbalance within our bodies. Despite noteworthy advancements in diagnosis and treatment in recent years, progress remains sluggish, and the projected death toll is set to reach a staggering 29 million worldwide by 2040 [1]. This trend is expected to worsen in the future, owing to the general population aging [2].

The conventional approach to treating cancer involves surgical removal of tumors, followed by radiotherapy and adjuvant chemotherapy, which should ideally target only malignant cells with high specificity. A drug may have broad targets, such as DNA replication or nucleotide synthesis, since malignant cells divide more quickly than normal cells, or it may have some degree of specificity when targeting extra- or intra-cellular protein networks.

These protein networks may belong to metabolic or protein–protein interactions (PPI). As oncogenesis can be viewed as a deregulation of the protein network that governs a cell, identifying molecular mechanisms driving oncogenesis and cancer progression is a crucial step toward developing more effective therapies. However, even with this perspective, the use of specific drugs for cancer treatment is challenging since complex real-world networks contain feedback loops that help maintain communication integrity if a vertex fails [3]. Consequently, malignant cells may resist a drug if an alternative metabolic or signaling pathway is available to complement the inhibited pathway. In the case of PPI networks, the inhibition of a particular vertex by a drug may have no effect on the network’s overall performance. Conversely, targeting hub vertices in a scale-free network is expected to fragment it into multiple components and ultimately lead to cell lethality. Thus, targeting vertices with a high connection degree and betweenness centrality is expected to increase the success rate of drug-inhibitory effects on malignant cells in cancer treatments [4].

Over the past thirty years, numerous molecular targets related to cancer (oncotargets) have been identified, and therapeutics have been developed to target them. However, many of the drugs currently available for specific cancers are quite costly, provide only moderate improvements in overall survival, and come with significant negative side effects. Indeed, the existing chemotherapies exhibit a wide range of acute and long-term side effects that can have substantial harmful consequences for patients’ health, mainly due to their low level of molecular specificity.

Given this situation, there is an urgent need for new strategies, models, or paradigms to critically evaluate molecular targets that may bring benefits to patients. These targets can be hubs involved in cancer maintenance through positive feedback loops [5] or involved in the physiological deregulation leading to cancer and metastasis development. Addressing this challenge is, in fact, the crux of personalized medicine today.

After about 40 years of investigation, PPI have been described to a level that is now adequate for modeling complex molecular processes, such as those involved in cancer. Traditionally, protein interactomes have been determined using experimental techniques, such as yeast two-hybrid, affinity pull-down mass spectrometry, and biochemical techniques, which information has been made available through online databases. More recently, progress in in silico data mining and high-throughput data generation related to gene, protein, and metabolic networks [4,6] has provided a new, very promising opportunity to identify proteins that may have marginal implications in normal cells but become signaling hubs in cancer cells due to their high natural rate of connectivity with other proteins and significant modifications in expression rates.

Complex networks are present in various fields, including physics, biology, and social sciences. A network can be described mathematically as a directed or undirected graph *G* = (*V*, *E*), consisting of vertex and edge sets *V* and *E*, respectively. An edge is included in the graph if there is a known interaction between the two partners, either by direct binding or enzymatic catalysis. Real networks exhibit a modular structure where vertices are organized into communities that are tightly connected internally and loosely connected to each other [7]. This structure results in the presence of symmetric subgraphs, such as trees and complete cliques [5], which help classify the vertices of a network into a backbone (those that remain fixed under automorphisms) and appendages (those that get mapped to other vertices).

Investigation into PPI modeling for cancer treatment [4,6] have shown a negative correlation between tumor entropy (measured through the distribution of connections in PPIs represented as oriented graphs) and the probability of 5-year overall patient survival (based on data from the Surveillance Epidemiology and End Results database—SEER) for certain types of cancer [6]. The complexity of the network was quantified through the use of Shannon entropy and betweenness centrality. Formula (1) can be used to formulate the complexity of a graph in terms of network entropy.

(1) $$H=-\sum_{v=1}^{n-1}p\left(v\right)logp(v)\;$$

where *p*(*v*) is the probability associated with a vertex *v*. Following Formula (2), the betweenness centrality coefficient *C_B_*(*v*) of vertex *v* can be calculated by counting the number of shortest paths between any two vertices in the network (denoted *s* and *t* and running through all pairs of vertices):

(2) $$C_{B}\left(v\right)=\sum_{s,t\in V}^{}\sigma \left(s,t|v\right)/\sigma (s,t)$$

where *σ*(*s*, *t*) is the number of shortest paths between these two vertices (*s*, *t*), *σ*(*s*, *t*|*v*) is the number of those paths passing through vertex *v* connecting the (*s*, *t*) pair, and *V* is the ensemble of *v* vertices of the network [6].

## 2. Gene Expression and Protein Network Hubs

We initially hypothesized that diseases with more resilient networks would exhibit greater resistance to drug treatment and would have a poorer prognosis.

**Figure 1:**
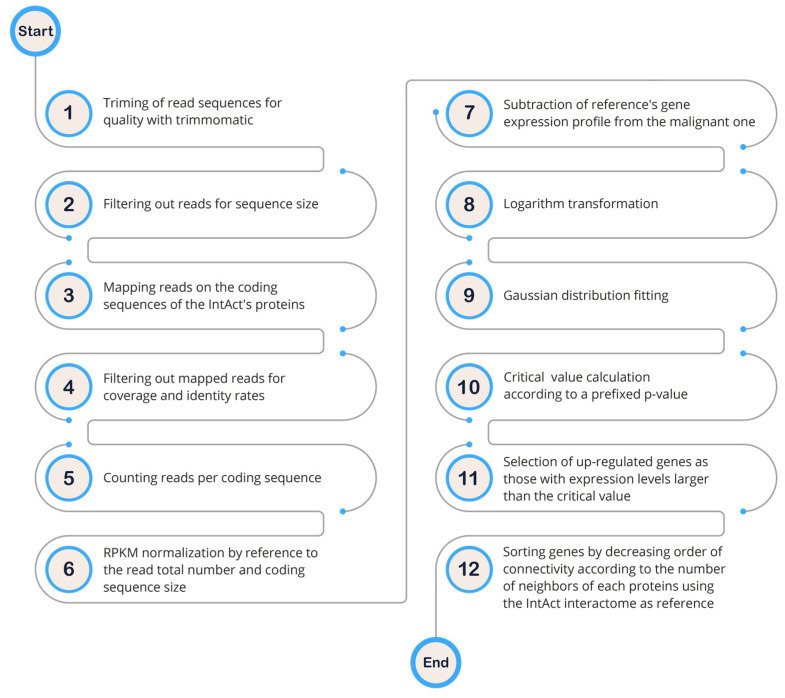
Schematic diagram illustrating the hub diagnosis from RNA-seq data.

To gain a deeper understanding of the mechanisms underlying solid tumor signaling pathways, we analyzed gene expression patterns across several breast cell lines to create cell-line-specific networks. Given the high correlation (r = 0.91) between betweenness centrality and protein connectivity (the number of E per V), we focused solely on protein connectivity. We searched for differentially expressed protein hubs in various malignant breast cell lines (Luminal A: MCF-7, T-47D, ZR-75-1; Luminal B: BT-474; Triple Negative: BT-20, MDA-MB-231, MDA-MB-468) compared to a non-tumoral cell line (MCF10A). The rationale for this approach is that drugs targeting up-regulated proteins are expected to have fewer side effects than those targeting non-differentially expressed proteins [8]. To achieve this, we obtained a subset of human genes with available interactome data by comparing human coding sequences (CDS) to protein sequences in UniprotKB. By mapping transcriptome reads from the aforementioned cell lines onto the human interactome CDS, we obtained the corresponding gene expression profiles. These profiles (read count per gene) were normalized using the reads per kilo base per million mapped reads (RPKM) method (Formula (3)) based on CDS size and total read count.

(3) $$RPKM=(10^{9}.C)/(N.L)$$

where 10^9^ is a correction factor, *C* is the number of reads that match a gene, *N* is the total mappable reads in the experiment, and *L* is the CDS size [9]. After subtracting the expression profile of the control cell line from those of the malignant cell lines, we observed symmetrical distributions of negative and positive values around zero. Taking the ${log}_{10}(x_{i}+1)$ of this distribution resulted in an approximately normal distribution, which enabled us to distinguish down- and up-regulated genes using a two-tailed *p*-value of 0.05% (Figure 1). 

As outlined in [10], apart from the conventional stages of a pipeline designed for the diagnosis of up-regulated genes, it is important to highlight here that a significant deviation in our approach lies in the evaluation of gene up-regulation. Specifically, our method involves a statistical comparison of a gene’s expression to the broader population of other genes, as opposed to a traditional gene-by-gene evaluation.

We discovered many hub proteins with a high degree of connectivity that showed no difference in expression between the malignant cell lines and the healthy control. This finding confirmed that a high degree of connectivity alone is not enough to design a drug treatment that minimizes side effects for patients. Interestingly, we did not observe any correlation between protein connectivity and gene expression (r = 0.1). Conversely, proteins with high connectivity that are up-regulated in malignant cell lines are promising targets for drug development.

**Figure 2:**
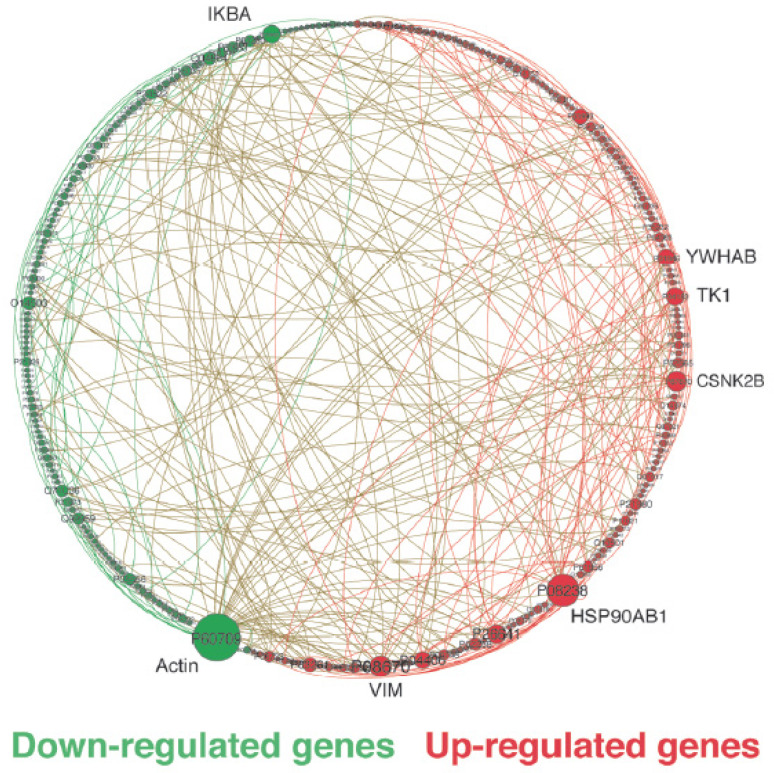
Circular layout of a sub-network consisting of differentially expressed genes between MDA-MB-231 (Triple-Negative) and MCF10A (non-tumoral). The nodes represent genes, while the links represent interactions between genes. We only clearly display the significant differentially regulated hubs with their gene symbols. Other vertices should not be taken into account and are shown according to their UniprotKB accession number. The size of the nodes corresponds to the degree of connectivity, and the colors (green for down-regulated and red for up-regulated) indicate the differential expression pattern of genes in tumoral versus non-tumoral breast cell lines. The network was visualized using Gephi (adapted with permission from ref. [8], Copyright is licensed under an open access Creative Commons CC BY 4.0).

Protein hubs refer to proteins (vertices) in a protein (or gene) network that have a much larger degree (number) of connections (edges) than the average values. They function as global regulators or integrators for multiple signaling pathways. Figure 2 provides a map of network interactions between down- and up-regulated genes in malignant breast cell lines, indicating that the top five most connected genes in sub-networks of up-regulated genes could be potential targets for drug treatment and/or development [8].

To identify potential targets for drug development in breast cancer, our investigation focused on malignant up-regulated genes coding for hub proteins. Through this approach, we discovered oncotargets (top-n genes) associated with key hallmarks of cancer, such as cell cycle control, resistance to cell death, induction of angiogenesis, invasion and metastasis, deregulation of cellular energetics, genome instability and mutation, and tumor-promoting inflammation, as defined by Hanahan [11]. Specifically, in the triple-negative subtype and luminal A, a higher percentage of up-regulated genes were found to be associated with sustaining proliferative signaling, resistance to cell death, and activation of invasion and metastasis, while in luminal B, up-regulated genes were preferentially associated with induction of angiogenesis, resistance to cell death, and activation of invasion and metastasis.

In triple-negative tumors, which are generally associated with poor prognosis, we observed the up-regulation of genes involved in various functions, such as (i) cell cycle control, including *EGFR*, *YWHAB*, *MAGOH*, *EEF1G*, *CSNK2B*, *TK1*, and *CHD3*; (ii) anti-apoptotic factors, such as *YWHAB* and *HDGF*; (iii) activation of invasion and metastasis, such as *YWHAB*, *CSNK2B*, *CHD3*, and *HDGF*; and (iv) angiogenesis, including *HSP90AB1*. These functions are all associated with the hallmark of tumor progression and support the poor prognosis of triple-negative tumors.

The sub-networks of up-regulated genes in luminal A cell lines indicated a function associated with tumor-promoting inflammation (*KPNA2*). Furthermore, transcripts overexpressed in luminal A cells were related to cell cycle control, including *GRB2*, *EEF1G*, *MCM7*, *CSNK2B*, *PAK2*, *TK1*, and *NPM1*, as well as *ERBB3*, *PAK2*, *TK1*, and *NPM1*, involved in cell resistance to death. Genes related to tumor progression included *HSP90AB1*, *GRB2*, *ERBB3*, and *CSNK2B*. The sub-networks in luminal B cells were similar to those in luminal A cells, as they were grouped under the luminal category. A comparison of luminal A and B cell lines with triple-negative tumors showed up-regulation of *ERBB2/3*, which evades growth factors in luminal A, and *YWHAB* and *PA2G4* in luminal B, implicated in cell resistance to death, sustaining proliferative signaling, and activation of invasion and metastasis. In summary, these results demonstrated the complexity of signaling through these networks and the massive consequences of protein hub deregulation on the crosstalk between regulators of cellular events.

## 3. Network Entropy and Breast Cancer Therapy

We applied the principles of systems biology described above to optimize the composition of drug cocktails in the context of personalized cancer treatment. We used network entropy as a quantitative measure, based on Shannon’s definition, to characterize the complexity of PPI networks [4]. Our goal was to evaluate the potential benefit of patient survival associated with the inactivation of the top-five protein hubs in up-regulated genes. We found that the proportion of total entropy represented by the top-five hub proteins is approximately 2% of the total protein network, on average. In the case of breast cancer, targeting these top-five hub proteins is expected to increase the 5-year survival rate for the majority of patients to over 100%, potentially improving the 10-year survival expectancy or even contributing to a permanent cure. Based on these findings and the availability of approved inhibitors on the market, we proposed several optimized drug combinations [12].

The drugs’ GI50 values and target annotations for the cell lines under investigation were obtained from [13]. The selected 74 drugs target a wide range of processes involved in cancer biology and progression and can be divided into two categories: (i) agents that target specific receptors (*n* = 54), including angiogenesis, cell cycle, microtubule/cytoskeleton, EGFR/FGFR/HER2/IGFR signaling pathways, Ras-Raf-MEK-MAPK-ERK pathway, mTOR pathway, PI3K-AKT pathway, HDAC epigenetic agents, and HSP90 targets; and (ii) cytotoxic chemotherapeutics (*n* = 20), including nucleotide synthesis, metabolism, DNA crosslinker, and multiple targets. Thus, we investigated the correlation between the GI50 of both target-specific and cytotoxic drugs and the entropy per node of the protein network in the control MCF10A cell line, as well as in luminal A (MCF-7, T-47D, ZR-75-1), luminal B (BT-474), and triple-negative (BT-20, MDA-MB-231, MDA-MB-468) malignant cell lines. Our findings indicate that malignant cell lines are generally more sensitive to target-specific drugs than to broadly cytotoxic ones. In contrast, cytotoxic drugs performed poorly, with their associated log10(GI50) never exceeding 5.3, on average, which is considered the minimal GI50 necessary to consider a candidate molecule a potential lead compound in state-of-the-art drug development. In contrast, target-specific drugs, on average, exhibited a log10(GI50) larger than the 5.3 threshold. When comparing the entropy per node of a cell line’s total protein network to its log10(GI50) value for different drugs, we observed a noisy pattern in cytotoxic drugs, whereas a clear negative correlation (r = −0.859) was evident, on average, for target-specific drugs. The negative correlation was particularly pronounced in luminal (r = −0.923) and, to a lesser extent, in triple-negative (r = −0.725) cell lines. Given that target inactivation through specific drugs appeared to be a more productive strategy than cytotoxic compounds, we listed the top five most connected proteins encoded by up-regulated genes according to a *p*-value of 0.05%. Subtracting the entropy contribution of each of the top five proteins from the total protein network entropy of the cell line under investigation gave us a net entropy corresponding to that network, which is equivalent to the benefit that can be expected from target inactivation. Interpolating with the orthogonal regression line (y = −0.000545267405602977x + 11.4731985709748) through the average network entropies of triple-negative, luminal, and control cell lines (11.4332400, 11.4294941, 11.4150921, respectively), on one hand, and patient 5-year survival (70, 90, 100, respectively [14]), on the other hand, most top-five targets’ inactivation brings the entropy back to values close to or lower than the control entropy [12].

As a result, the top-n targets merit consideration for drug development as they have the potential to provide a complete 5-year overall survival (OS) for the patient population under consideration.

It is worth noting that the current drug libraries have a limited availability of compounds that target specific proteins. Hence, the use of drug combinations offers a greater benefit in chemotherapy compared to treatments that rely on a single drug.

For instance, the drug fusicoccin is anticipated to exhibit effectiveness against the majority of breast cell lines, as its protein target is typically among the top up-regulated hub proteins, with the exception of MCF-7. However, if the aim is to enhance patient survival over a period of ten years, it would be advisable to combine fusicoccin with other drugs based on the specific case.

## 4. In Vitro Validation of Hub-Based Theranostics

Theranostics is an emerging field of medicine that integrates targeted therapy with specific diagnostic tests. This patient-centered approach represents a transition from conventional medicine to a contemporary personalized and precise approach. The newest theranostics paradigm employs nanoscience to unite diagnostic and therapeutic applications into a single agent, enabling diagnosis, drug delivery, and treatment response monitoring. Theranostic agents can circulate systemically, bypass host defenses, and deliver diagnostic and therapeutic agents to the target site, allowing for cellular and molecular-level diagnosis and treatment of the disease [15].

Any new health technology must go through the steps of pre-clinical and clinical validation to be accepted by bioethics committees and medical advisory boards. Pre-clinical validation involves in vitro and in vivo testing, while clinical validation typically involves two clinical trials. The first trial is limited to 60 people, and if the results are conclusive, a statistical assessment is performed on a larger patient cohort. The purpose of these validations is to demonstrate that the new technology can provide a substantial benefit to patients compared to existing technologies, thereby enabling physicians to take the risk of using it. The risk is that a physician who chooses the new technology may stop using the well-established one, and if the new technology is not as successful as anticipated, the physician’s choice could end up harming the patient.

**Figure 3:**
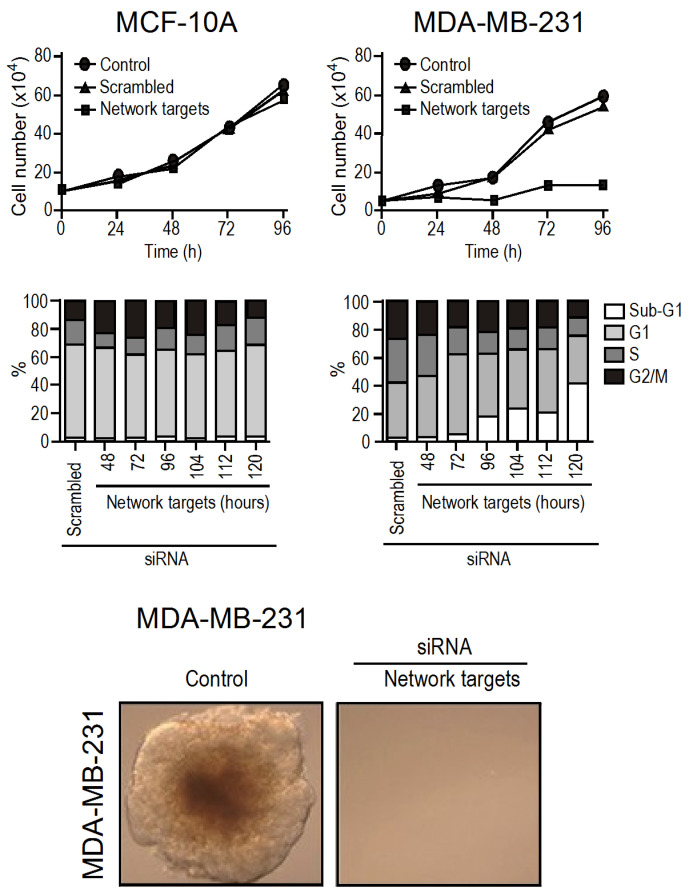
In vitro validation of the hub-based theranostic concept. In comparison to MCF10A, the top five up-regulated hubs in MDA-MB-231 were *HSP90AB1*, *CSNK2B*, *TK1*, *YWHAB*, and *VIM*. When these five mRNA were silenced simultaneously through siRNA interference, the growth of MDA-MB-231 was halted while that of MCF10A remained unaffected (**top panel**). The increase in cell death was observed by 48 h (white rectangles) as depicted by flux cytometry (**middle panel**). Furthermore, the metastatic potential was eliminated (**bottom panel**) (adapted with permission from ref. [16], Copyright is licensed under an open access Creative Commons CC BY 4.0).

Up to this point, we have achieved successful in vitro validation of hub-based theranostics through small interfering RNA (siRNA) and found it to be conclusive. By selecting the top-five targets that were up-regulated in the triple negative MDA-MB-231 cell line compared to the non-tumoral MCF10A, we observed that MCF10A continued to grow normally while the growth of MDA-MB-231 was stopped, and the metastatic potential of MDA-MB-231 was completely turned off. Cell death was observed to increase from 48 h to 120 h (limit of the experiment) after silencing (Figure 3).

Interestingly, siRNA silencing is transient, and its effect vanishes after 48 h. Thus, our findings indicate that network disarticulation shows some level of inertia, but when the process starts, it appears to be irreversible. We also observed that silencing only one target at a time did not have any effect on the MDA-MB-231 growth or viability. Therefore, the combined effect of silencing all five targets together was greater than the effect of silencing them separately [16].

## 5. How Many Targets Should Be Inhibited?

Due to the fact that primary tumor material is a more promising resource for developing targeted therapeutic approaches compared to malignant cell lines, we conducted an analysis on a cohort comprising RNA-seq data from 475 paired samples of the TCGA database (https://portal.gdc.cancer.gov/, accessed on 21 October 2018), which is renowned as a standard reference for conducting tumor analysis through RNA-seq [10,17]. Interestingly, [18] reported that RNA-seq from stroma is equivalent to that of healthy tissue in matter of control representativity for malignant tissue comparison. This finding allows one to catch a glimpse of clinical application using stromal tissue from biopsy or surgical pieces as a control to assess the profile of malignant gene regulation. 

These analyses revealed PPI hub targets that are overexpressed in 70% to 100% tumors of a given cancer type (one-size-fits-all hub targets) and others that are much more personalized. This observation underscores the significance of certain hubs in sustaining a particular cancer and highlights the necessity to identify these hubs (akin to the requirement for mutation detection) to attain a deeper comprehension of the patient’s condition [10].

**Figure 4:**
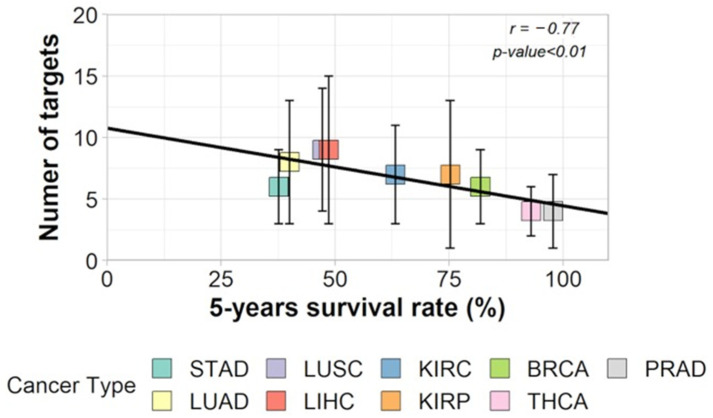
Correlation between number of targets and 5-year OS of each cancer type (Stomach adenocarcinoma: STAD, Lung adenocarcinoma: LUAD, Lung squamous cell carcinoma: LUSC, Liver hepatocellular carcinoma: LIHC, Kidney renal clear cell carcinoma: KIRC, Kidney renal papillary cell carcinoma: KIRP, Breast invasive carcinoma: BRCA, Thyroid cancer: THCA, Prostate cancer: PRAD) (adapted with permission from ref. [17], Copyright is licensed under an open access Creative Commons CC BY 4.0).

With a TCGA cohort of RNA-seq samples covering 475 patients through nine different cancer types, we confirmed that the RPKM normalization method, together with the use of the IntAct interactome (https://www.ebi.ac.uk/intact/home, accessed on 1 January 2017), resulted in a larger entropy drop under specific attacks against hubs, as predicted by Albert et al. [3]. After validating this data treatment, we confirmed the negative correlation between entropy and 5-year OS [17]. In this experiment, we updated the coding sequence number corresponding to the human IntAct proteins to approximately 15,000 (75% of the human proteome) CDS and approximately 150,000 interactions. Despite the data heterogeneity (different tumors, sequencing laboratories, sequencing technologies, and technical teams), the coefficient of negative correlation between the average degree entropies per cancer type and cancer aggressiveness (measured through the statistics of 5-year overall survival—OS) was surprisingly high: r = −0.68 [17]. By “average degree entropies”, we mean the average entropy of the PPI network of up-regulated genes for each tumor of a given cancer type. The relationship between degree entropy and aggressiveness of tumors served as a base for the following reasoning by Conforte et al. [17]: (i) Since the correlation is close to 0.7, it was reasonable to fit a linear regression (y = −0.004x + 2.507) through the negative relationship of cancer average entropy and cancer aggressiveness, and (ii) 100% survival correspond to a given entropy threshold; thus, for a given tumor entropy, one might measure the number of hubs that should be removed from the network to decrease its entropy to the threshold of 100% 5-year OS. If we use drugs for these targets, this process tells us which drugs and how many of them should be used to increase the patient’s 5-year OS close to 100%. On average, the number of targets to be inhibited was between three for lowly aggressive cancers (thyroid, prostate) and ten for highly aggressive ones (lung, liver, stomach), as per [17] (Figure 4).

The methodology described in [17] has been automated [10], which has revealed that certain targets, including HSP90AB1, are more conserved across various tumors and cancer types than others. This is not surprising given that cancer is a genomic disease characterized by numerous mutations, somatic crossovers, translocations, and other karyotype abnormalities [19], which leads to a decline in protein functionality due to protein folding issues. One-way malignant cells adapt to this is by overexpressing chaperones to facilitate proper protein folding. Conserved protein targets across various tumors and cancer types align with the one-size-fits-all concept, which seeks single treatments that can control tumors in large patient cohorts for a given cancer type. The one-size-fits-all approach justifies the use of cytotoxic drugs that target broad molecular processes, such as those involved in cell division control. However, the efficiency of such treatments for patients whose tumors do not respond to them is uncertain, as these patients may become weakened by the treatment and eventually succumb to its consequences. This is likely the main argument for developing a personalized approach to oncology that results in rational chemotherapy by applying a treatment designed for each patient’s tumor molecular phenotype. While some argue that personalized cancer treatment is more expensive for society, reducing hospitalization time may have a favorable impact on total cancer costs [20].

The use of drug cocktails containing a larger number of drugs is increasingly common in routine clinical practice, and these can be administered intravenously [21]. The optimal sequence for administering such cocktails is still being analyzed worldwide. It is worth noting that the disarticulation of the malignant signaling network through specific attacks against protein hubs is cumulative but has an initial inertia, as observed by Tilli et al. [16]. Henceforth, the therapy involving the targeted administration of medications against specific protein hubs may be employed in conjunction with the presently endorsed cytotoxic-based treatment regimens. This type of drug combination would likely improve patient outcomes, as described above.

## 6. Markers of Aggressiveness

The hallmark of cancer that corresponds to deregulated cell proliferation is a critical feature [11] and is accountable for the harmful effects on patients. It is caused by the disruption of various pathways, including those involved in growth factor signaling, developmental pathways, such as WNT, Hedgehog, Notch, and Hippo, and other mechanisms, such as mTOR and NF-κB signaling that affect cell growth and proliferation [22]. Additionally, the interplay among these pathways in cancer must be considered [23]. For example, some tumors display interactions between the WNT pathway and other important ones, such as EGF, FGF, Hedgehog, mTOR, Notch, TGF-β, and NF-κB [24]. Crosstalk can alter the expected functions of individual pathways, resulting in a more intricate and complex network that is difficult to predict.

**Figure 5:**
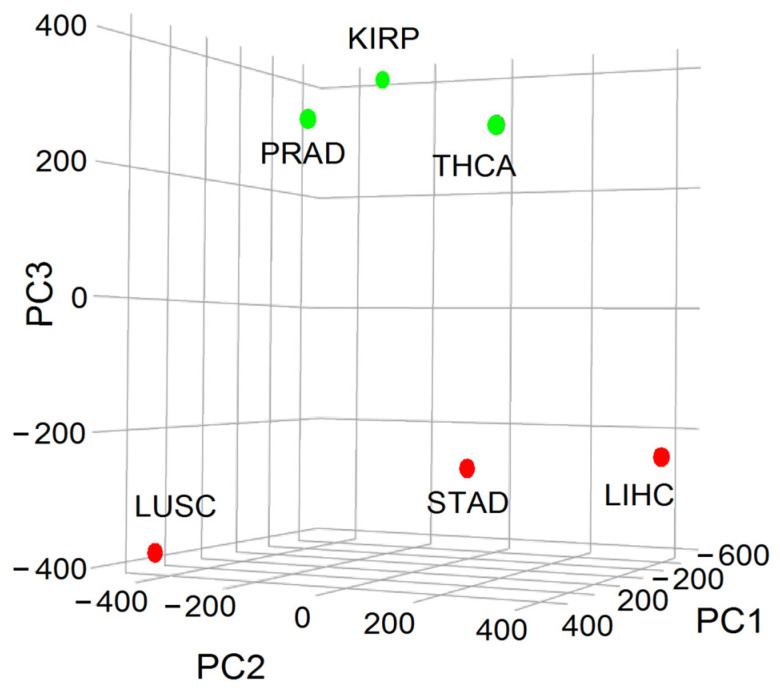
Principal component analysis (PCA) representation of the variance associated with cancer aggressiveness considering the WNT and cross-linked pathways showing a clear division of cancer types into H (red: STAD: stomach adenocarcinoma, LUSC: lung squamous cell carcinoma, LIHC: liver hepatocellular carcinoma) and L (green: KIRP: kidney renal papillary cell carcinoma, THCA: thyroid cancer, and PRAD: prostate cancer) classes according to PC3 (adapted with permission from ref. [23], Copyright is licensed under an open access Creative Commons CC BY 4.0).

As previously mentioned, the inverse relationship between entropy and 5-year OS made it possible to classify cancer aggressiveness into two distinct groups: the high (H) class, comprising tumors from lung, liver, and stomach cancers, and the low (L) class, comprising tumors from renal papillary, thyroid, and prostate cancers. By approximating entropy using the relative frequency of malignant up-regulated genes from WNT and interconnected pathways weighted by their connection rate in the IntAct interactome, we employed principal component analysis (PCA) to demonstrate that 19% of the variance is attributable to aggressiveness. This proportion was linked to the third component (PC3), which effectively differentiated H and L classes (Figure 5).

As a result of our analysis, we have identified the genes that best explain the variance between the H and L classes, namely *CTNNB1*, *SKP1*, *CSNK2A1*, *PRKDC*, *HDAC1*, and *YWHAZ*. These genes are all hubs and were expressed at higher levels in the H class than in the L class. Interestingly, when we used a random forest classifier (RFC) to classify tumors as belonging to either the H or L class, the list of genes was different and included *CAD*, *PSMD14*, *APH1A*, *PSMD2*, *SHC1*, *TMEFF2*, *PSMD11*, *H2AFZ*, *PSMB5*, and *NOTCH1*. Although this discrepancy may seem strange, it is because the PCA algorithm identifies the genes that best explain the variance between the H and L classes, while the RFC algorithm maximizes the classification power of a given tumor as belonging to either the H or L class. Additionally, when we mapped the genes listed by both PCA and RFC onto the interactome, we found that they were all close neighbors [23].

## 7. Modeling Cancer Dynamics

Due to the modular nature of real networks, the molecular signaling pathways of tumors possess redundancy in their connections [7]. Therefore, a significant question that remains unanswered in the approach described by Carels et al. [8] is which of two up-regulated targets in a tumor with an equal or similar degree of connectivity would be more appropriate for silencing to maximize the benefit of a patient? Would it be more efficient to inhibit vertices in one or multiple pathways (modules)? Answering this question is still essential for developing rational personalized therapy based on hub silencing. The functional effects of inhibiting hubs on signaling cascades and whether it is more effective to inactivate hubs that belong to the same or different modules will be studied using RNAi silencing.

### 7.1. Boolean Networks

The Boolean network is a method for modeling the dynamic behavior of malignant networks and identifying the attractors that define them. In the first step of modeling breast cancer, the vertices and topology of a Boolean network were defined based on the subnetwork of up-regulated genes from RNA-seq data of the MDA-MB-231 cell line, as described previously. By utilizing single-cell RNA-seq (scRNA-seq) and interactome data, the dynamics of malignant subnetworks of up-regulated genes were studied through Boolean canalyzing functions. The binarized version of scRNAseq data was employed to identify attractors that were specific to each patient and critical genes related to each subtype of breast cancer [25]. These attractors were characterized by genes reflecting the tumor subtypes of each patient, consistent with the personalized oncology concept. Then, the model was investigated to detect critical genes involved in malignant attractor stability whose inhibition could optimize the induction of tumor cell death and serve for potential applications in cancer theranostics [26].

### 7.2. Hopfield Networks

**Figure 6:**
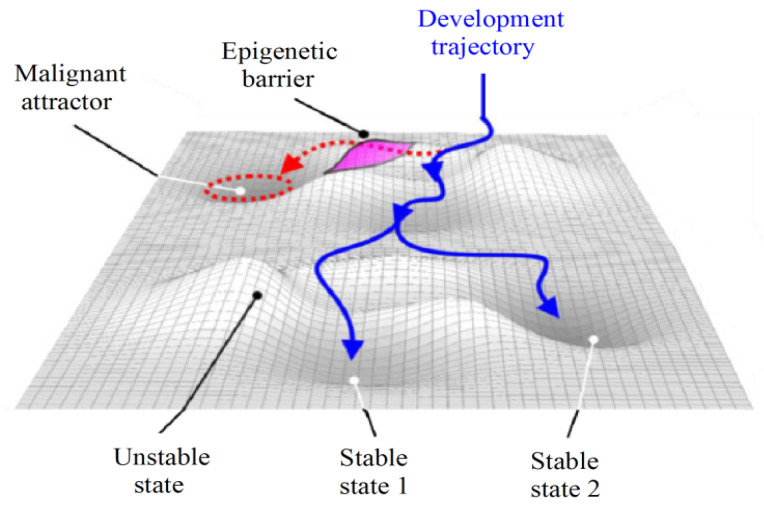
Epigenetic landscape and attractors (adapted with permission from [30]).

Modeling the basins of attraction can aid in comprehending the molecular behavior of tumors [27]. In this regard, each gene expression profile corresponds to a cell state in a multidimensional space where stable states define cell phenotypes and are characterized as equilibrium points in the phase space. The stochastic nature of gene regulation causes other states to result in the same attractor phenotype and, thereby, to contribute to its basin of attraction [28,29]. These features are analogous to the Waddington epigenetic field, where the valleys depict the basins of attraction of the attractors and the hills represent epigenetic barriers and unstable states (Figure 6).

**Figure 7:**
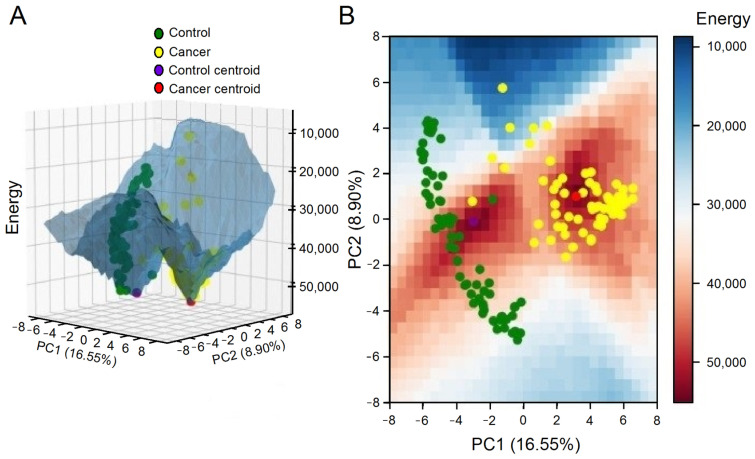
A three-dimensional (**A**) and a two-dimensional grid (**B**) were used to plot an energy landscape depicting both control and tumor attractors, as well as samples (adapted with permission from ref. [27], Copyright is licensed under an open access Creative Commons CC BY 4.0).

In some instances, a phenotype may transition towards another, such as when normal cells convert into malignant ones due to the vanishing of an epigenetic barrier between two attractors caused by genetic alterations or mutations [31]. Huang et al. [28] suggested that a cancer attractor would be confined within epigenetic barriers that would prevent its emergence. However, as a cell accumulates mutations and experiences gene deregulation, these epigenetic barriers are lost, making the cancer attractor attainable (Figure 7).

The evidence suggests that the epigenetic landscape is not fixed and can undergo changes over time [28,32]. In light of this, Ao et al. [32] proposed a classification system for cancer based on its functional landscape, which distinguishes between preventable, curable, and incurable cancers. For example, our analysis revealed that the greater the Euclidean distance between a tumor sample and the control attractor, the worse the patient’s overall survival [27].

Due to the above-mentioned reasons, inhibiting genes that belong to the same basin of attraction as the malignant state may aid in disrupting it. Since cancer is a result of attractors in the cellular dynamics’ phase space, cancer therapy should redirect the signaling network towards a new basin of attraction, inducing active cell death [28,29]. The analysis of the signaling network state space could aid in identifying the basin that corresponds to the desired attractor, which would allow the optimization of the number of targets recommended for treatment. Moreover, this strategy could also assist in determining the order of priority for therapeutic interventions, i.e., the shortest trajectory in the phase space required to achieve the basin of attraction associated with the desired state [29], i.e., malignant cell death.

### 7.3. Ergodicity and Cancer

The inherent temporal constraint involved in probing gene expression phase space reduces the capacity of data obtained to portray a comprehensive representation of cellular states. This limitation is particularly acute during the progression of cancer, where the lengthy temporal scope of malignant transformation [33] hinders the data’s capacity to model the full breadth of cellular states. A potential solution to this impasse is to employ the concept of ergodicity, which is associated with the aggressiveness of tumors. This methodology enables bridging scRNA-seq data with the theoretical underpinnings of system dynamics, thereby offering a statistical framework for data characterization [34].

Ergodicity is a statistical property that describes how the system explores the phase space over time [35] and could help study the statistical properties developed by cancer cells. Moreover, in an ergodic system, the period to explore the different configurations is of the order of an external (experimental) observation time. In biological terms, this means studying how the system visits all molecular phenotypes that characterize each tumor stage over time. Additionally, the statistical properties measured from a single trajectory of the system can be considered representative of the entire ensemble in an ergodic system. Regarding gene expression, the absence of stringent constraints in malignant cells—as compared to healthy cells—culminates in highly heterogeneous observations over a truncated temporal scale, as displayed by disparate scRNA-seq profiling. In this way, taking the average trajectory of these different phase space states allows a representative description of the system.

Instead of envisaging ergodicity over the entire gene expression configuration space, another plausible strategy involves invoking the concept of internal ergodicity [36]. The distinction between full ergodicity and internal ergodicity lies in the scope of exploration. Full ergodicity implies that the entire system explores all possible states, whereas internal ergodicity focuses on the ergodic behavior of individual components within the system, i.e., on the basins of attraction in the epigenetic landscape. A system can have internal ergodicity without full ergodicity, meaning that while the individual components explore their respective phase spaces completely, the overall system may not explore all possible states. Thus, according to this definition, internal ergodicity may represent a given stage characterized by its attractor related to its specific centroid of gene expression. This proposition understands the gene expression configurations to be navigated as constrained portions of the space, such as areas associated with cancer attractors. Adopting an internal ergodicity premise enables the interpretation of centroids derived from gene expression data clustering techniques as near-equilibrium states. This interpretation simplifies the process of parameter estimation and the examination of deterministic and stochastic dynamics, thereby facilitating the exploration and calculation of the (epi)genetic landscape of various tumor subtypes without necessitating time series data [34].

**Figure 8:**
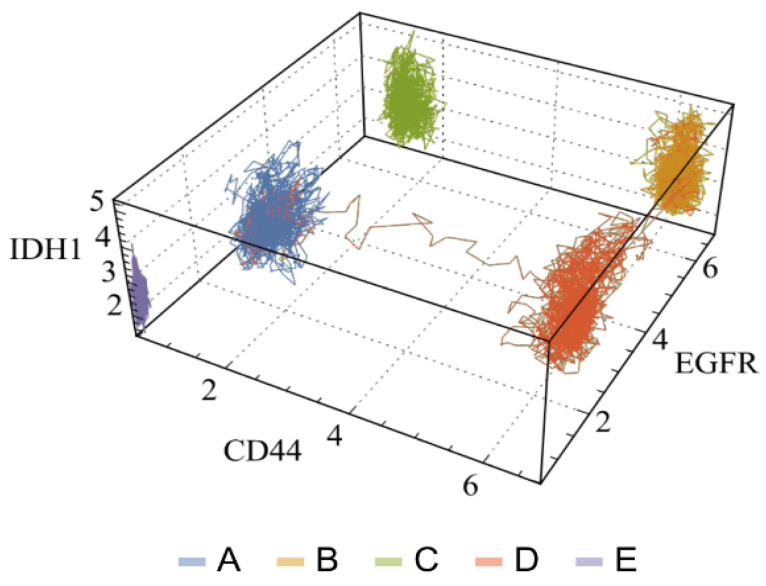
Three-dimensional visualization of full trajectories in a three-subtype marker space of glioblastoma multiforme. Each axis shows the expression values of a specific marker gene, while each line corresponds to the complete time of the three trajectories analyzed. Each color or letter denotes the corresponding basin (adapted with permission from ref. [34], Copyright is licensed under an open access Creative Commons CC-BY-NC-ND 4.0).

By leveraging internal ergodicity, Vieira et al. successfully obtained significant alignment between experimental and simulation-derived data centroids [34]. The study also assessed the in silico stability of each cluster by (i) determining transition time, (ii) analyzing time series autocorrelation, and (iii) contrasting the equivalence of time and sample averages. This method enabled a more intricate understanding of the system’s dynamics and presented a novel way to quantify internal ergodicity. Notably, the study revealed transitions between attractor basins (Figure 8), accounting for rare events that minimally affected the internal ergodicity premise.

These transitions between attractor basins suggest a potential interaction among cancer subtypes, a phenomenon that could be fundamental to cancer recurrence. However, it is important to acknowledge that factors like genetic mutations and epigenetic regulations, which contribute to temporal variations in gene expression, are only approximated as constants in short-term characterization. However, this approximation could provide valuable insights into tumor progression when investigating parameter time changes linked to a transient state landscape [34].

In summary, the ergodicity paradigm posits that identifying components that may prompt the system to shift from non-ergodic to internally ergodic or to fully ergodic states could elucidate the mechanisms of cancer progression, potentially informing the development of therapeutic interventions.

It is worth noting that therapy-oriented clinical trials based on Hopfield, Boolean, and (epi)genetic landscape modeling are still lacking. However, the effort of modeling integration into clinical pipelines is ongoing [37,38,39,40,41].

## 8. Drug Repurposing

The process of developing new drugs has some drawbacks, including its multistep nature and the significant time and cost involved. Clinical trials are often unsuccessful in the initial phases, making the process even more challenging. One way to address these limitations is through drug repurposing, also known as drug repositioning, which involves using a drug for a therapeutic application other than its original indication [42]. Repurposing non-oncology small-molecule drugs has become an increasingly attractive approach to improving cancer therapy, as it may result in lower overall costs and shorter timelines. Various non-oncology drugs approved by the FDA have recently been reported to treat different types of human cancers [43]. Traditionally, drug repurposing was typically performed by in vitro screening of compound libraries [44]. A more recent approach is in silico compound screening. 

The purpose of computer-aided drug design and screening is to reduce the number of compounds that need to undergo in vitro validation. This rapidly evolving field has numerous advantages, such as (i) the availability of more than 190,000 protein structures in the Protein Data Bank (PDB) for most clinically significant targets or their close orthologs, complemented by recent advances in high-resolution cryo-EM, which can now resolve large proteins and complexes that were previously inaccessible by NMR or X-ray diffraction; (ii) chemical libraries have become increasingly diverse and expanded to include hundreds of millions, or even billions, of virtual compounds based on optimized click-like chemistry that is Readily Available (REAL), i.e., they can be synthesized in as little as 3 to 4 weeks with an 80% success rate; and (iii) the accuracy and reliability of structure-based virtual screening methods continue to improve regularly and provide a 10%–40% routine hit rate (see refs in [45]).

The chaperone protein HSP90AB1 (Hsp90β) presents a promising prospect for drug repurposing, primarily owing to its (i) central role as a hub in signaling pathways, (ii) involvement in the stabilization and transportation of various proteins, and (iii) elevated expression across a wide range of cancer types. Consequently, inhibiting HSP90AB1 has a significant impact on numerous biological functions [46].

Due to its toxicity, geldanamycin is currently avoided as an inhibitor of Hsp90β [47]. Therefore, we conducted an investigation of FDA-approved drugs to identify a suitable substitute candidate. Employing an integrated approach encompassing molecular modeling, molecular docking, and molecular dynamics, we identified ritonavir as a promising drug candidate to augment the arsenal against cancer. Ritonavir has previously been suggested by [48] as a potential drug for clinical investigation due to its low toxicity and potential inhibition of Hsp90β.

Ritonavir, an antiretroviral drug used for HIV infection treatment, functions as a protease inhibitor [48]. It binds to proteins belonging to the HSP90 family and partially inhibits their chaperone function [49]. Additionally, ritonavir inhibits the proteasome [50,51] and induces stress on the endoplasmic reticulum. Furthermore, ritonavir facilitates the inhibition of Akt binding to HSP90, resulting in Akt dephosphorylation and inactivation [48,52]. Ritonavir exhibits a Kd value of 7.8 µmol/L within a clinically achievable concentration range. These characteristics likely contribute to the inhibition of proliferation in the malignant cell line MDA-MB-231, given its sensitivity to Hsp90β inhibition by RNAi [16]. Although the mechanism of HSP90 inhibition by ritonavir remains unclear [53]. However, interaction between ritonavir and Arg382 has been predicted to play a critical role in stabilizing the closed conformation of Hsp90β required for ATPase activity [54]. Therefore, the interaction of ritonavir with Arg382 may elucidate its inhibitory effect on HSP90s.

## 9. RNA Interference

Most of the hub targets are derived from the signaling pathways and are not amenable to drug intervention. Consequently, drug repurposing is challenging. As an alternative approach, cyclic peptides have been proposed [55]. They offer improved stability compared to linear peptides and possess larger molecular sizes than small molecules, thereby increasing the probability of protein interactions.

In an alternate approach, siRNA can be used to inhibit gene expression since every disease-related gene has the potential to be targeted by siRNA based on its base sequence alone [56]. While significant progress has been made in the clinical application of RNAi therapy over the past two decades, naked siRNA can easily trigger an innate immune response and be degraded by ribonucleases. Additionally, due to their negative charge and large molecular weight, siRNAs have limited permeability across the cellular membrane, necessitating the utilization of a carrier system to facilitate their intracellular delivery [57].

Initially, viral vectors were used for siRNA delivery due to their efficacy in promoting gene expression. However, this approach has inherent drawbacks, including potential carcinogenic effects, mutations, and immunogenicity. Furthermore, scalability poses a challenge in utilizing viral-based strategies [58,59]. As a result, alternative non-viral approaches have emerged for siRNA delivery, such as the utilization of lipids and polymers. Lipids, in particular, have found wide application in this context, with the first FDA-approved medication, patisirian, relying on lipid nanoparticles for the treatment of hereditary transthyretin-mediated amyloidosis [60].

Alongside patisiran, the FDA has granted approval for four other formulations. Givosiran has been approved for the treatment of acute hepatic porphyria, lumasiran for primary hyperoxaluria type 1, inclisiran for primary hypercholesterolemia, and vutrisiran for hereditary transthyretin-mediated amyloidosis [61,62,63,64]. These formulations have also received approval from the Brazilian Health Regulatory Agency (Anvisa), except for inclisiran, which is currently undergoing evaluation [65]. While only a limited number of formulations have been approved for commercialization, there are many ongoing studies aiming to develop siRNA-based strategies [66,67,68,69].

Achieving the therapeutic effects through siRNA therapy entails a significant challenge in ensuring their efficient delivery to the intended targets. siRNAs must overcome various barriers, including internalization by phagocytic cells, degradation by nucleases, rapid elimination through the kidneys, and liver excretion [70,71]. The obstacles encountered in siRNA delivery may differ according to the specific targets being addressed. However, critical factors contributing to these challenges include the hydrophilic nature of siRNA molecules, their polyanionic nature, high molecular weight, insufficient cellular uptake, and the need to escape from endosomes [71,72].

Nanoparticles, however, have great potential as carriers for effective siRNA delivery into malignant cells. A notable example is the development of DCR-MYC, a lipid-based siRNA nanoparticle designed to downregulate the oncoprotein MYC, which is implicated in the development of various malignancies. Treatment with DCR-MYC resulted in tumor shrinkage in multiple patients, demonstrating its therapeutic effectiveness [73]. These results highlight the promise of nanoparticle-based RNAi technologies in cancer treatment [74]. Combining nanoparticle technology with hub-based theranostic would provide an ideal approach to rational cancer therapy.

Chitosan, a widely used biopolymer in medical applications, such as in the development of nanoparticles for therapeutic agent delivery, possesses several appealing characteristics. These features include its abundance in nature, biocompatibility, biodegradability, and modifiable structure. Various cross-linking methods can be used to create chitosan nanoparticles, such as precipitation, ionotropic gelation, reverse micellar, and emulsion coalescence [75]. Moreover, due to its cationic nature, chitosan serves as an effective vector for nucleic acids [76,77]. When exposed to acidic pH, the amino groups within chitosan structure undergo protonation, resulting in a positive charge at pKa around 6.2. This feature enables chitosan to form polyplexes with siRNA molecules, which possess negative charges on their phosphate groups [77,78]. The application of chitosan nanoparticles for siRNA delivery has been investigated for the treatment of various types of cancer, and recent studies have shown promising results with respect to chitosan-based nanoparticles [79].

In a study conducted by Liang et al. [80], a mouse model of bladder tumor with CD44 overexpression was targeted by siRNAs encapsulated in chitosan nanoparticles conjugated with hyaluronic acid dialdehyde. Both in vitro and in vivo experiments exhibited significant efficiency in encapsulating siRNA with minimal cytotoxicity. Furthermore, the nanoparticles demonstrated effective targeting of the CD44 receptor and facilitated the release of siRNA into the T24 malignant cells via a ligand-receptor-mediated mechanism. This process showcased the nanoparticles’ capability to disrupt the BCL2 oncogene in vivo.

Hajizadeh et al. [81] devised a siRNA delivery system using thiolated chitosan and trimethyl chitosan-based nanoparticles decorated with TAT peptide to enhance its uptake by cancer cells. The primary aim of this investigation was to target hypoxia-inducible factor (HIF) due to its direct association with a tumor’s oxygen deficiency and its role in stimulating the expression of the CD73 enzyme. Both HIF and CD73 are implicated in tumor vascularization, altered metabolism, and increased proliferation of cancer cells. The developed system exhibited promising outcomes, demonstrating a significant reduction in cell migration and proliferation. This study suggested that the simultaneous silencing of CD73 and HIF-1α could serve as a potential strategy for effectively suppressing tumor growth.

Zhang et al. [82] formulated nanoparticles based on chitosan and hyaluronic acid to explore the potential of siRNA delivery in tumors of lung cancer, among the most aggressive worldwide. In the investigation, the nanoparticles were loaded with cyanine-3-labeled siRNA and evaluated on A549 cells derived from non-small cell lung cancer. The results demonstrated that the nanoparticles successfully released the siRNA by interacting with the CD44 receptor and effectively hindered cell proliferation through the downregulation of BCL2 gene. Those results highlight the potential of engineered nanoparticles to enhance targeted siRNA release.

It is imperative to address the inherent challenge associated with reaching specific target cells in the lungs by combining various nanoparticle characteristics. Therefore, controlling the size and distribution of nanoparticles, incorporating suitable functional groups on their surface, and optimizing the capacity for therapeutic agent release are crucial factors to consider. Additionally, ensuring high degradability and low toxicity are essential prerequisites in the design of these nanoparticles [83].

Iron oxide (Fe_3_O_4_) and gold (Au) nanoparticles have emerged as promising vehicles for siRNA delivery, drawing significant research attention. Some studies have demonstrated the Au nanoparticles’ potential to surpass critical obstacles associated with siRNA therapy, such as immune system activation and unintended transfection of healthy cells. Moreover, the carriage of siRNA with Au nanoparticles could protect against enzymatic degradation, facilitating effective mediation of gene silencing. However, the use of metal nanoparticles needs more exploration of certain aspects. These include addressing concerns about cytotoxicity and nonbiodegradability, requiring meticulous investigation. Furthermore, a comprehensive understanding of the modulation of cellular responses is imperative to establish the safety and reliability of metal platforms for siRNA delivery [84,85]. Nonetheless, the properties of nanoparticles may be altered to align with the biological system. Cellular uptake of nanoparticles is essential for increased efficacy, and some studies have shown that for Au nanoparticles, the uptake depends on surface functionalization [84]. Baghani et al. [86] developed Au nanoparticles modified with trimethyl chitosan for the delivery of siRNA in the treatment of breast cancer. The use of chitosan was proposed for the increase in cellular uptake and stability. The system demonstrated a significant knockdown (86%) of epidermal growth factor receptors. In addition, Au nanoparticles have been employed in the delivery of siRNA associated with drugs with the aim of finding a more effective strategy for the treatment of cancer. For example, a multifunctional system based on Au and doxorubicin for the codelivery of siRNAs has been developed by Tunç and Aydin [87].

In addition to their use in solid tumors, nanoparticles can represent a revolution in leukemia treatment specificity. Hematological malignancies are great candidates for this approach. A simple blood sample (liquid biopsy) can give us access to the entire molecular profile of the patient’s tumor, providing the information needed for more personalized therapeutic regimens. Unlike solid tumors that require nanoparticles to reach the site of action, liquid tumors are distributed throughout the circulatory system. Therefore, the barriers that nanoparticles need to overcome to reach solid tumors are less critical in leukemias; although the access of therapeutic sites, such as the bone marrow and lymphoid tissues, can be more difficult [88]. Despite the fact that nanoparticles can be opsonized by blood proteins and recognized by the mononuclear phagocytic system [89], the addition of specific antibodies targeting membrane proteins can be a solution for nanoparticles to reach cancer cells, deliver their content, and reduce their toxicity [90]. The use of nanoparticles has been tested for the treatment of leukemia in several preclinical studies [88,91,92,93]. Although there are still many problems to solve, such as specificity, long-term toxicity, and clearance, this technology has the potential to reinvent cancer treatment.

However, RNAi is not the only avenue when specific drugs are unavailable; other technologies are emerging. For example, BioNTech has successfully created a range of mRNA-based vaccines, exemplified by the authorized COVID-19 vaccine Comirnaty. This achievement substantiates the efficiency, stability, and practicality of employing this technology. It also underscores the viability of utilizing nanoparticles to deliver RNA interference (RNAi) for disrupting protein–protein interaction (PPI) hubs. Concurrently, alternative methodologies like CRISPR are also advancing towards personalized tumor treatments, as evident from recent reports [94,95,96].

## 10. Cancer-on-a-Chip for Personalized Treatments

The use of tumor organoids as a preclinical reliable model system is already a reality [97,98,99]. Although their ability to retain characteristics of native tumors in comparison to cell monolayer models, they are unable to mimic mechanical and fluid-flow dynamics of a patient’s body. In this sense, the convergence of organoids and microfluidic cell-based devices have led to the development of physiologically relevant tumor microenvironments within biochips [100,101]. The manufacture of these microfluidic chips relies on different microfabrication techniques in transparent material, allowing the real-time observation of cells [102].

Organ-on-a-chip technology has the potential to overcome key challenges in traditional in vitro culture and animal testing, especially for applications of personalized treatment strategies and drug screening [100,103]. Within micron-sized channels, organoids of different cell types can be culture in a controlled dynamic microenvironment, providing several benefits over static cell culture, such as tissue-like construct with cellular and morphological fidelity as well as continuous nutrient supply and waste removal [101,104]. Moreover, it is possible to engineer microfluidic systems to mimic signaling cascades in continuous-flow cell culture, raising information to elucidate signaling pathways and multi-tissue crosstalk [103].

In the area of cancer research, one major promise of the so-called “cancer-on-a-chip”, or “tumor-on-a-chip”, is that they can help us understand the role of mechano-stimuli in cancer development and metastasis [105,106]. Mechanical forces play a crucial role in cancer pathogenesis, especially involving the metastasis biomechanics scenario. The malignant progress involves a complex interplay of both acellular and cellular factors, including extracellular matrix, nutrient levels, and immune cells [105]. The pathological biomechanical conditions that contribute to the development and progression of cancer are frequently characterized by three factors: (i) uncontrolled proliferation of tumor cells within a confined space, resulting in mechanical stress on the surrounding tissue; (ii) increased deposition of extracellular matrix components, which leads to abnormal tissue stiffness; and (iii) altered mechanical forces within the tumor microenvironment that facilitate cancer cell migration and invasion [101,105]. For instance, using patient-derived tumor biopsy samples, a personalized “tumor-on-a-chip” device could enable real-time monitoring of the anticancer efficacy of adoptive T cell immunotherapy and the responses of resident lymphocyte populations [107]. 

Several features of cancer-on-a-chip platforms have paved the way for personalized treatment options of patient-derived cells models, facilitating the identification of patient-specific drug sensitivities, boosting personalized medicine and drug development [104]. By fluidically connecting fluids flowing through different organoids—such as the liver, kidney, and intestine—it is possible to systematically assess a drug candidate’s biological response, potentially revealing any associated side-effects [101,108]. Although animal models may still be required to investigate drug disposition in the whole body and other effects, the use of multi-organ systems may be the most advanced tool to assess multi-organ physiology and pathology in vitro. Physiological responses of the entire body can be simulated in vitro through the utilization of patient-derived tumor biopsy samples [104], enabling the obtention of personal information in a more accurate way.

## 11. In Vivo Validation

Establishing a correlation between experimental animal models and their applicability for clinical translation is a significant hurdle in precision medicine. This is frequently achieved through the screening of thousands of molecular targets in in vitro cultures, ex vivo tissues, or animals, to capture the response of a complex system over time and constructing predictive models of human physiology to facilitate the development of clinical trials [109,110].

Preclinical validation can be achieved by cultivating individual tumors in vitro to determine the safest and most effective treatment before administering it to the patient. In vitro cultures of tumor organoids have shown a 90% consistency of somatic mutations and DNA copy number profiles with the patient’s original biopsies or surgical samples [111]. However, the genomic evolution of implanted cells from patient-derived xenograft models may be altered due to differences in the microenvironment and physiology of mouse tissues, leading to therapy response variations when compared to humans [112,113]. Other challenges impeding the widespread use of patient-derived xenografts include the length of time required to generate each model, the difficulty in analyzing results due to the need to use immune-compromised mice, the low success rate of xenografting, the significant resources required to create and maintain these models, as well as the lack of standardized clinically relevant criteria [110].

As for xenografts, validating human tumors in vivo presents the challenge of their rejection by the immune system of the animal host, which recognizes them as foreign due to the restriction to major histocompatibility tissue (MHC) antigens. To address this issue, immune-deficient mice, such as nude, SCID, NOD, NOD-SCID, NOG, and NSG mice, which lack innate and/or adaptive immune systems, can be used. However, this approach is still limited since it does not take into account the role of the immune system, which is a significant factor for primary tumor microenvironment uniqueness and metastatic spread. 

A potential solution to these issues is to use a mouse model that possesses an intact immune system and to use RNAi to knock down up-regulated genes coding for hubs in malignant cell lines of mice, as described above for human tumors. To maintain consistency with previous work, we are currently performing this exercise on the 4T1 triple-negative spontaneous breast cancer metastasis model, generated through orthotopic transplantation of 4T1 tumor cell line into immunocompetent BALB/c mammary gland [114,115]. Unlike most tumor models, 4T1 tumor cells can spontaneously metastasize from the primary tumor to multiple distant sites, including lymph nodes, blood, liver, lung, brain, and bone, in a way very similar to that of human breast cancer [114,115].

## 12. Clinical Trial

The notion of precision oncology remains to be fully tested and various approaches for its implementation are still in need of assessment [116]. Within the oncology community, there is significant debate surrounding the merits and future of precision oncology, primarily due to its frequently overstated advantages [117]. Currently, clinical trials aimed at validating the efficacy and usefulness of biomarkers and omics tests are not generally yielding successful results.

The primary clinical trial methodologies utilized to evaluate precision oncology include (i) umbrella trials, which comprise a master protocol wherein patient eligibility is determined by the existence of a tumor type that is sub-stratified based on particular molecular alterations linked to distinct anticancer therapies [118], and (ii) basket trials, which involve patients with various tumor types that share a common molecular alteration treated with a corresponding matched therapy [119,120]. Both methodologies are categorized as platform trials, as they enable the assessment of multiple hypotheses within a single protocol, resulting in expedited results and reduced costs [114]. Another example is the Octopus trial, which specifically investigates various drug regimens, often in combination with a single backbone drug [121].

Due to its multidimensional nature, the validation of precision oncology through clinical trials may require the use of adaptive design, as proposed by Garralda et al. [122]. Precision oncology is inherently multidimensional for several reasons, including (i) the dependence of drug combination on the molecular profile of the patient’s tumor, (ii) the influence of the patient’s genetics on drug metabolism, and (iii) the multifactorial and unpredictable dynamics of the disease as reported in medical records. Adaptive models, which are typically Bayesian, aim to enhance the performance of the classifier by increasing the weights of reliable predictors and decreasing the weights of unstable predictors while selecting the best therapy based on current patient characteristics.

Adaptive designs, which rely on real-time clinical outcomes, facilitate dynamic trials by promptly discontinuing ineffective arms and increasing patient randomization to evaluate more effective treatments and improve biomarker selection. As a result, these trials necessitate a reduced number of participants and shorter follow-up periods compared to traditional randomized trials [121].

The enhanced flexibility offered by complex designs is accompanied by increased complexity, which brings certain limitations. The interpretation of results obtained from intricate designs may be affected by a higher risk of type I errors, leading to reduced reproducibility and potential rejection of regulatory approval. To enhance the reliability of these complex designs, it is crucial to provide detailed documentation regarding parameter specification, operating characteristics, experimental design, statistical analysis, and user-friendly software [123].

Next-generation precision medicine trials encompass various approaches, such as the I-PREDICT and WINTHER trials, which focus on individualized treatment for each patient in a unique manner, following the N-of-1 type design [121,124,125]. In N-of-1 trials, the outcomes are typically compared with historical or real-world outcome data, as well as with results from higher or lower degrees of matching. The objective is to optimize treatment selection, including customized combinations, by considering the specific characteristics of the tumor and the patient, with the aim of enhancing treatment efficiency. However, the limitations of this type of trial include the absence of a reference standard, the heterogeneity of treatments, and the complexity of the analyses required to identify the optimal treatment [121].

Additionally, home-based trials have emerged as a strategy to facilitate drug access for patients who are unable to travel and participate in traditional site-based clinical trials. This innovative approach facilitates patient recruitment and enrollment rates by allowing their active engagement in the trial from the comfort of their homes [121]. Patients are under the care of an extensive network of investigators and home-health nurses who are responsible for ensuring the patient’s well-being. A significant portion of the recruitment and monitoring activities are conducted remotely. However, there are potential drawbacks, such as suboptimal monitoring of adverse events and timely assessment of treatment response [121].

Reconciling the patient-centric model of precision medicine with the necessary regulatory authorization for the introduction of new drugs or therapeutic approaches presents several challenges. One of these challenges involves accurately identifying molecular changes that are drivers of disease as opposed to transient ones. It is crucial to develop strategies that enable efficient and reliable utilization of various designs and the vast amount of available data. This will facilitate rapid and productive advancements in the field, ultimately maximizing the benefits for patients afflicted by cancer diseases.

If a hub-based theranostic approach is addressed, a potential solution would be to initially restrict clinical trials to hub targets that are associated with approved drugs. In this way, the clinical trial would solely need to assess the efficacy of the computational method employed to diagnose the up-regulated hubs. As the drugs have already been approved and their potential interactions described, the remainder of the inquiry has already been validated. Consequently, patients should be categorized based on the up-regulated hubs corresponding with the existing set of approved drugs, which would serve as an intermediary model between umbrella and basket trials. In this context, we believe that N-of-1 trials would be the most suitable approach for hub-based theranostics.

## 13. Discussion

As for any target, up-regulated protein hubs can be more or less personalized from one tumor to the other. Hub overexpression may result from the mutation of other genes rather than from the gene under consideration or gene from copy number multiplication. The tumor landscape undergoes constant alteration, with the emergence of new cell lines due to secondary mutations. Nevertheless, the pivotal focus is on how these changes in the gene expression landscape [126] impact cell homeostasis. It is these changes in the gene expression landscape that affect gene regulation through retroactions via feedback loop in the PPI network (neglecting ncRNA action in this system) [127]. The critical notion pertains to the following: Within a disease, there exist primary (pathogenicity factor) and secondary (virulence factor) determinants [128]. The primary determinants encompass factors that facilitate the disease (tumor) to establish itself within the host (human body), whereas secondary determinants pertain to the disease’s virulence (level of aggressiveness). Every system is characterized by both its external (environmental) and internal attributes. Consideration can be given to epigenetic initial conditions (internal factors distinct from mutations) or chemical conditions (external factors influenced by pollution or agricultural chemicals, distinct from mutations) that could alter the gene regulation landscape in a manner that triggers the onset of the cancer process [126]. Depending on the molecular attributes of the biological system under investigation, cancer onset can be dominated or not [129]. Consequently, a continuous selective pressure from the environment affects a particular system, influencing its survival within that environment [130,131]. As a result, the primary factors must be upheld to sustain the tumor within its surroundings. This situation aligns with the presence of a malignant basin of attraction that involves genes crucial for tumor viability [27]. However, the imperative for specific genes to be overexpressed for tumor survival need not be exclusively dependent on the occurrence of point mutations within their sequences [132]. Instead, one must focus on the factors that are essential to sustain the system. For instance, if the survival of the tumor hinges on the elevated expression of the chaperone HSP90β, it does not inherently indicate a mutation within this gene. Instead, a process of negative selection would operate to prevent mutations in this gene, ensuring its functional integrity. In essence, this signifies that a functional HSP90β is requisite to accurately fold mutated proteins that play a pivotal role in the cancer’s persistence.

Our findings have significant implications for cancer therapy, including (i) providing a strategy for identifying potential oncotargets for a given tumor; (ii) revealing the critical regulatory circuits between up-regulated and down-regulated genes that contribute to the physiological progression of tumors; and (iii) facilitating the rapid identification of protein targets for personalized medicine that can be tailored to individual tumor types and histological subtypes. The methodology we present can establish a quantitative relationship between oncotargets and a rational therapeutic strategy. Our study provides a framework for identifying key players involved in malignancies caused by solid tumors, which could lead to new insights for the development of therapeutic interventions for cancer treatment and prevention. However, further research is necessary to functionally validate these oncotargets, starting with pre-clinical testing at an in vivo level.

The presence of an interdependent sub-network among down- and up-regulated genes suggests that the differentially expressed genes, besides being stimulated by particular cancer pathways, also interact with each other in a compensatory manner. This observation further supports the notion that oncogenesis and tumor progression involve intricate and multi-faceted signaling mechanisms. The network structures identified for various cell subtypes and their corresponding patterns conform well to the existing literature.

With the rapid progress of modern technology, it is foreseeable that shortly, when a patient is diagnosed with cancer, both malignant and peripherical cells from biopsies or surgical pieces would be sequenced to inform the treatment plan. Upon identifying specific oncotargets, it would become theoretically possible to tailor a personalized drug combination based on existing knowledge or biopharmaceuticals, as suggested by the RNAi approach adopted by Pfizer and BionTech. Theoretically, this strategy can be largely automated and compatible with personalized medicine. However, as response rates to a specific chemotherapeutic drug may be relatively low in an unselected pre-treated patient population, it is necessary to pre-select those patients with a favorable molecular profile in their malignant cells, i.e., those with the highest likelihood of benefiting from the treatment. 

Our strategy differs from traditional drug repurposing by seeking new indications for cocktail therapies that target essential pathways/mechanisms resulting in cancer cell death with minimal side effects on normal cells, aiming to maximize efficacy and minimize toxicity. This strategy is expected to overcome intrinsic and acquired resistance, tumor heterogeneity, adaptation, and genetic instability of cancer cells. However, multiple alternative signaling routes in tumors can make them resistant to drug treatment, so several issues must be addressed to ensure that the proposed strategy is as effective as predicted, as biological complexity can always lead to unexpected situations.

As mentioned above, the hub-based approach offers a methodical strategy for cancer therapy. Nonetheless, numerous challenges still need to be addressed in order to attain a rational approach to cancer therapy. For instance, it is likely that factors beyond the degree of hub connectivity need to be considered. Addressing these factors may necessitate the utilization of mathematical and computational modeling [133]. Mathematical methods and hypotheses, as well as computational modeling to describe PPI and gene regulatory networks, are also constantly improving [134,135,136,137,138]. The philosophical underpinning of how the transition from a normal state to a malignant state can be accurately described in mathematical terms is a subject that merits contemplation [139,140,141]. Furthermore, there is a gradual integration of other molecular entities, such as non-coding RNAs (ncRNAs), into these networks [142,143]. Hallmarks and pathways possess distinct implications for tumor survival, and the selection of targets among them can potentially influence the outcomes of therapeutic interventions [11,144]. The dynamics of malignant basins of attraction and the critical genes that disrupt them to drive the tumor towards cell death are contingent upon these variables [139,145]. When contemplating the therapy itself, there arises the question of how to best administer the treatment for a specific array of targets that require inhibition. Should it be carried out sequentially? If so, what sequence and dosages should be employed? In addition, hub-based therapy can be applied in complement to classical chemotherapy with the purpose of maximizing benefits to patients. Addressing such queries can be facilitated through control theory (reverse engineering), leveraging existing pharmacological data [146,147,148,149,150]. In closing, it is evident that mathematical and computational modeling will progressively emerge as integral elements of clinical care, particularly in the context of cancer, attaining elevated levels of sophistication in the foreseeable future [38,151].

The principle of maximizing patient benefit depends on identifying protein targets that serve as connectivity hubs in the signaling pathway and are up-regulated in malignant cells while minimizing harmful side effects for the patient’s health, which is crucial for their well-being and long-term survival after treatment. When the benefit of a 5-year OS exceeds 100%, it implies that the benefit should be assessed in terms of longer-term survival, such as 10-year survival, which is currently the benchmark for evaluating breast cancer outcomes. However, determining the optimal drug cocktail to maximize 10-year survival and minimize harmful side effects on patients, remains challenging at this time. Employing recent advances in cancer-on-a-chip technologies, the evaluation of personal drug cocktails could also be performed with patient-derived tumor cells, which could raise information about signaling pathways and multi-tissue crosstalk within an in vitro dynamic microenvironment. 

The use of integrated methods of bioinformatics, computational biology, and artificial intelligence in medicine and healthcare is a rapidly growing field and is anticipated to provide benefits to patients, healthcare professionals, governments, and insurance companies. The research presented in this study aligns with this trend in that rational chemotherapy has the potential to reduce patient suffering, enhance diagnostic accuracy, and reduce costs, all of which would be advantageous for society as a whole. However, any innovation carries with it clinical, social, and ethical risks, and careful consideration must be given to assess the comparative advantages of the strategy outlined here with existing methods, as outlined by Lekadi et al. [152]. In that respect, the participation of clinical and pharmaceutical entities is critical but difficult to obtain.
